# Investigating drug-liposome interactions using liposomal electrokinetic chromatography

**DOI:** 10.1007/s00216-025-05783-6

**Published:** 2025-02-13

**Authors:** Alice Šimonová, Martin Balouch, František Štěpánek, Tomáš Křížek

**Affiliations:** 1 Department of Analytical Chemistry, Faculty of Science, Charles University, Hlavova 8, Prague 2, 128 00 Czech Republic; 2 Zentiva, K.S., U Kabelovny 130, Prague 10, 102 37 Czech Republic; 3 Department of Chemical Engineering, University of Chemistry and Technology, Technická 5, Prague 6, 166 28 Czech Republic

**Keywords:** Active pharmaceutical ingredients, Capillary electrophoresis, Interactions, Liposomal electrokinetic chromatography, Liposomes, Pseudostationary phase

## Abstract

This study explores the potential of using liposomal electrokinetic chromatography as a ranking method for the rapid and simultaneous evaluation of drug-membrane interactions of a larger group of substances and assessing their sensitivity to tissue-specific parameters, namely pH, temperature, and lipid composition. We used a group of nine model drug substances to manifest how molecules could be classified for the relative sensitivity of drug-membrane interactions to pH and temperature. We observed that increasing the amount of liposomes in the background electrolyte significantly affected the separation kinetics of various active pharmaceutical ingredients, altering their mobility and/or peak shapes. Experiments with liposomes from bovine liver and heart tissue extracts revealed different interactions based on the lipid composition. Canagliflozin, which initially showed no electrophoretic mobility, migrated toward the anode in the presence of negatively charged liposomes. Mobility of positively charged substances, ambroxol and maraviroc, was suppressed by the interactions with liposomes. Their peaks also exhibited significant tailing. The effect on the separation of negatively charged compounds was significantly weaker. A small change in mobility was observed only in the case of deferasirox. We also examined the effect of temperature during separation, and we observed that increased temperature generally enhanced effective mobility due to lower electrolyte viscosity and increased lipid bilayer fluidity. Lastly, we tested the effect of sodium phosphate buffer pH (ranging from 6.0 to 8.0) with 4% liposomes on drug-liposome interactions. However, the effects were complex due to changes in API ionization and liposome surface charge, complicating the distinction between pH effects and liposome presence on API behavior. Our findings emphasize the significance of liposome composition, temperature, and pH in studying the interactions of liposomes with drugs, which is crucial for optimizing liposome-based drug delivery systems.

**Supplementary Information:**

The online version contains supplementary material available at 10.1007/s00216-025-05783-6.

## Introduction

The interaction between drugs and lipid membranes is crucial both for the understanding of phenomena occurring in living systems such as pharmacokinetics [1], biodistribution, and drug permeation [2] and for technological applications such as drug encapsulation to artificially made lipid vesicles [3]. A number of experimental methods have been developed for studying drug permeation and partitioning into lipid bilayers. These methods include classical permeation assays such as PAMPA [4], Franz cell permeation [5], cell line permeation Caco-2 [6] or MDCK [7], or BLM [8], as well as liposome-based assays that benefit from the significantly larger surface area available for permeation and can therefore capture even the kinetics of slowly permeating substances [9–11]. The above-mentioned methods are typically used for the measurement of drug-membrane interaction either for individual solutes or their binary mixtures with additives such as permeation enhancers [12–14]. In pharmaceutical development, the need arises to evaluate a larger number of molecules (e.g., drug candidates) simultaneously and to rank them according to their affinity towards the lipidic membrane as a function of tissue-specific parameters such as temperature, pH, or composition of the lipid mixture. In early drug development, the tested substances are often available only in small quantities [15, 16], and therefore, methods based on bulk solutions are not generally suitable.

Methods that enable the simultaneous measurement of drug-membrane interaction for a larger number of substances without their excessive consumption are based on chromatography that uses liposomes as the stationary phase [17]. The driving force can be either a pressure gradient [18, 19] or an electric field (electrophoresis) [20]. The latter method is particularly suitable for mixtures of substances that carry a different charge and thus influence the local force field as the membrane interface.

Liposomal electrokinetic chromatography combines elements from both capillary electrophoresis and chromatography. In this approach, liposomes are freely suspended within the background electrolyte (BGE), and they serve as a pseudostationary phase inside the capillary, facilitating chromatographic separation. Through a combination of electrophoretic mobility, electroosmotic flow, and chromatographic interactions with the liposomal phase, LEKC enables the study of API-liposome interactions under controlled electrophoretic conditions. Understanding these interactions is crucial for optimizing drug delivery systems, unraveling drug release mechanisms, and predicting in vivo behavior [21, 22]. Such interactions can influence the kinetics of analytical separations in various ways, including changes in mobilities or alterations in peak shapes. The utilization of CE with liposomes as a pseudostationary phase offers several advantages, such as enhanced separation efficiency and the ability to closely mimic the physiological conditions of biological membranes by varying the lipid composition. This approach not only deepens our understanding of drug-lipid interactions but also supports the development of more effective and targeted drug delivery systems. Numerous LEKC studies have been conducted [23] using different membrane compositions of liposomes: liposomes from natural sources [24], liposomes composed of common lipids such as POPC or DPPC [25, 26], or even compositions with ceramides to mimic skin behavior [27]. The tested molecules are mostly recruited from common drug molecules [28], but also studies specifically aimed at pesticides [29], hormones [30], or specifically charged molecules exist [26]. From the LEKC studies, different variables can be extracted, such as partition coefficient [26], binding constants [31], or even permeability [32]. Even though LEKC has proven a useful tool in modeling drug-membrane interactions, it should be noted that liposomal membrane is not an ideal model of a biological membrane and that LEKC provides an environment that differs from the one in a living organism, which leads to some principal limitations of this approach. First, the ionic strength of the physiological solution is impractical for BGE in LEKC because it exhibits very high conductivity, leading to high electric currents and overheating of the solution in the capillary. As the temperature has a significant effect on the measurement results and increased temperature can compromise liposome stability, such phenomena must be avoided by using BGEs of lower ionic strengths. Second, to observe the effect of API’s interaction with liposomes, the electrophoretic mobilities of free API and API-liposome complex must differ. The greater the difference the greater the observed effect. This can lead to an underestimation of the interactions between APIs and liposomes with the same sign of electric charge. Another point to be considered is the possible effect of applied voltage on the structure of the liposomal membrane and its interactions with APIs.

The above-mentioned studies mainly dealt with individual substances, and the LEKC set-up and experimental conditions were generally different in the individual studies. This makes the comparison of quantitative parameters such as effective mobility challenging. Therefore, in this work, we investigate the potential of using LEKC as a ranking method for the rapid and simultaneous evaluation of drug-membrane interaction of a larger group of substances with varying physicochemical properties and assessing their sensitivity to tissue-specific parameters, namely pH, temperature, and lipid composition. While previous studies have predominantly utilized simple lipid compositions, our work advances the field by incorporating complex, tissue-derived lipids that closely mimic in vivo conditions. A group of nine model drug substances was selected to represent a broad range of physicochemical properties (hydrophobicity, dissociation constants, charge) for a thorough evaluation. The therapeutic class, chemical class, or mechanism of action were not considered. Using this group of drugs, we propose that molecules can be classified for the relative sensitivity of drug-membrane interaction on pH and temperature, which may provide guidance for the expected behavior of the substance in vivo.

## Material and methods

### Chemicals and reagents

Thiourea, 99% was purchased from Sigma-Aldrich (Burlington, USA). Sodium dihydrogen phosphate dihydrate, p.a. was purchased from Lach-Ner (Neratovice, Czech Republic). Sodium hydrogen phosphate dodecahydrate, p.a. was purchased from Lachema (Brno, Czech Republic). Sodium hydroxide was purchased from Penta (Prague, Czech Republic). All standards of active pharmaceutical ingredients, namely ibuprofen, valsartan, febuxostat, canagliflozin, atorvastatin calcium, maraviroc, deferasirox, aprepitant, and ambroxol hydrochloride, were provided by Zentiva (Prague, Czech Republic). Methanol ≥ 99% was purchased from VWR International (Radnor, USA). All samples and background electrolytes were prepared using deionized water produced by the Milli-Q system from Millipore (Danvers, USA).

Lipids for preparation of liposomes, namely 1,2-dipalmitoyl-*sn*-glycero-3-phosphocholine (DPPC), 1,2-dipalmitoyl-*sn*-glycero-3-phospho-(1′-*rac*-glycerol) (sodium salt) (DPPG), liver polar lipid extract (bovine), and heart polar lipid extract (bovine), were purchased from Avanti Polar Lipids (Alabaster, USA). DPPC and DPPG were mixed in the 3:1 molar ratio. The composition of liver and heart polar extracts is shown in the Electronic Supplementary Material Table S1. All samples were prepared with a total lipid concentration of 5 mg/ml by lipid film hydration method [33]. Briefly, lipids were dissolved in chloroform/methanol mixture (*v*:*v*, 1:1). Then, the solution was placed on a rotary evaporator, and at constant temperature (60 °C), the pressure was lowered from atmospheric to approximately 100 mbar. After the complete evaporation of the solvent, samples were placed in a desiccator for at least 12 h. Sample rehydration was made in 10 mmol/l sodium phosphate buffer at pH 7.10. Prepared liposomes were extruded through a membrane with a pore size of 400 nm, and their size and zeta potential were measured using dynamic light scattering (Malvern Zetasizer NanoZS).

### Instrumentation

All experiments were performed on a 7100 CE instrument from Agilent Technologies (Waldbronn, Germany) equipped with a diode array detector. A fused-silica capillary was purchased from Polymicro Technologies (Phoenix, USA). For the measurement of pH, a 3540 pH/conductivity meter from Jenway (Staffordshire, UK) was used.

### Electrophoretic conditions

The inner diameter of the fused-silica capillary was 50 μm, the outer diameter was 375 μm, the total length was 50.0 cm, and the effective length was 41.5 cm. The capillary temperature was maintained at 25 °C unless stated otherwise. Before every set of measurements, the capillary was flushed for 10 min with 1 M sodium hydroxide and 10 min with deionized water. Before each run, the capillary was flushed for 2 min with the background electrolyte. Samples were injected hydrodynamically by a pressure of 5 kPa for 5 s. A separation voltage of 20 kV was then applied, and the electric current in 10 mM sodium phosphate buffer at pH 7.10 without the addition of liposomes was approximately 17 μA. UV detection at 200 nm was employed in all cases.

## Results and discussion

In this study, LEKC experiments were performed in BGEs with different amounts and compositions of liposomes, at different pH, and at different temperatures. To investigate the effect of individual variables on the API-liposome interactions, two parameters were evaluated from the LEKC electropherograms, i.e., migration time and USP tailing factor of each API peak. From the API migration time, the apparent electrophoretic mobility can be calculated. By subtracting the electroosmotic flow (EOF) mobility, calculated from the migration time of a neutral marker, the effective mobility of the API is obtained. If there is a quickly established equilibrium between free API and API-liposome complex, we cannot observe two separate peaks of API and API-liposome complex but rather one symmetric peak whose effective mobility is a linear combination of the electrophoretic mobilities of the two. The stronger the API-liposome interaction, the more the equilibrium composition is shifted towards the complex, and the observed effective mobility is closer to the one of the pure complex. Hence, strong API-liposome interactions will result in a peak with the effective mobility greatly differing from the mobility of free API, but with unchanged peak symmetry. On the other hand, if the kinetics of the interaction is rather slow, gradual association and dissociation of the API-liposome complexes while free API, free liposomes, and their complexes are migrating in different velocities, often even in different directions, will distort the peak shape. Hence peaks with similar mobility but altered symmetry after the addition of liposomes to BGE indicate a relatively strong interaction with slow kinetics.

### Liposomes from DPPC and DPPG

For the preliminary experiments, we used liposomes prepared from DPPC and DPPG lipids in a 3:1 molar ratio hydrated in 10 mM sodium phosphate buffer, pH 7.10. At first, four APIs were selected to test the effect of the liposome amount in the BGE on the separation kinetics. We used two APIs that have a positive charge (maraviroc and ambroxol hydrochloride), one neutral (canagliflozin), and one with a negative charge (deferasirox) under selected conditions. The electropherograms obtained with the increasing amount of DPPS:DPPG liposomes present in the BGE consisting of 10 mM sodium phosphate buffer; pH 7.10 can be seen in Electronic Supplementary Material, Fig. S1. The changes in the peak shapes may not be obvious from the figure, and mobility values do not directly correlate with migration times as the EOF mobility is involved. Thus, the corresponding effective mobility and USP tailing factor values are summarized in Table 1. Three of four APIs showed changes either in their mobility or peak shape with increasing amount of liposomes. Originally, canagliflozin (peak no. 3) did not exhibit electrophoretic mobility in the phosphate buffer without the presence of liposomes. When they were added into the BGE, canagliflozin migrated out of the neutral zone in the anodic direction. Its peak broadened with the increasing amount of liposomes keeping a symmetrical shape so the USP tailing factor did not change significantly, while its anodic effective mobility linearly increased. Positively charged maraviroc (peak no. 2) showed small changes in the peak shape as the USP tailing factor at 5% height increased about 1.5 times indicating occurring interactions with liposomes. Tailing of the peak, more pronounced with the increasing amount of liposomes, could indicate a slow kinetics of the interaction; on the other hand, the effective mobility of maraviroc remained unchanged. This would suggest that due to the slow interaction kinetics, only a small fraction of maraviroc molecules engaged in the interaction, which led to increased tailing but unchanged overall effective mobility. The second positively charged API, ambroxol hydrochloride (peak no. 1), showed a substantial change in the peak shape and also a significant decrease in its cathodic effective mobility. As little as 2% (*v/v*) of liposomes in the BGE increased the USP tailing factor at 5% height more than two times. With the increasing amount of liposomes, the peak broadened even more and with decreasing effective mobility it fully disappeared in the baseline. The dramatic change in the peak shape and significant tailing points to a relatively slow kinetics of the interaction. Unlike maraviroc, the effective mobility of ambroxol decreased to less than 80% of its original value in 6% (*v*/*v*) liposomes indicating a significant part of ambroxol molecules engaging in the interaction. Interestingly, the log P values (see Electronic Supplementary Material Table S2) suggest higher lipophilicity of maraviroc when compared to ambroxol. The higher lipophilicity of maraviroc might hinder the electrostatic interactions between negatively charged liposomes and positively charged API. On the other hand, the negatively charged deferasirox (peak no. 4) showed no changes in peak shape and/or effective mobility. This finding supports the importance of electrostatic interactions between API and liposomes. Nevertheless, it should be noted that due to the anodic effective mobility of deferasirox and the anodic effective mobility of liposomes, an interaction of the same strength will result in a significantly smaller change in the effective mobility than in the case of neutral or positive API. Overall, the experiments with DPPC/DPPG lipids showed that in this experimental setup, it is possible to observe interactions of the APIs with liposomes, which can manifest as changes in mobility as well as changes in peak shape, but these observations are limited by the rigidity of DPPC/DPPG membrane. Thus, we proceeded to experiments with more biologically relevant liposomes based on tissue extract lipids. These lipid extracts contain many lipids and the liposomes that can be produced from them will result in higher fluidity and therefore stronger possible interactions with APIs. Furthermore, this more fluid structure is native to body membranes, and thus, experiments with tissue extracts can better mimic the behavior of body membranes.

**Table 1:** Obtained electrophoretic mobilities *μ*_eff_ and USP tailing factor in BGE containing liposomes from DPPC and DPPG at total lipid concentration 5 mg/ml in 3:1 molar ratio. BGE: 10 mM sodium phosphate buffer, pH 7.10 with increasing amount of liposomes; temperature: 25 °C

% lip	*μ*_eff_ ⋅ 10^8^, m^2^ V^−1^ s^−1^				USP tailing (at 5% height)			
	Ambroxol	Maraviroc	Canagliflozin	Deferasirox	Ambroxol	Maraviroc	Canagliflozin	Deferasirox
0	1.57 ± 0.02	0.95 ± 0.02	-	− 1.73 ± 0.02	2.96	3.77	-	0.57
2	1.52 ± 0.01	1.01 ± 0.01	− 0.12 ± 0.00	− 1.71 ± 0.01	6.77	3.98	ND*	0.56
4	1.41 ± 0.01	1.01 ± 0.02	− 0.21 ± 0.00	− 1.72 ± 0.01	ND*	4.13	1.24	0.56
6	1.23 ± 0.03	1.00 ± 0.01	− 0.29 ± 0.01	− 1.72 ± 0.00	ND*	4.13	1.01	0.56
8	-	0.99 ± 0.01	− 0.38 ± 0.01	− 1.71 ± 0.00	-	4.96	1.03	0.57
10	-	0.99 ± 0.02	− 0.45 ± 0.01	− 1.72 ± 0.00	-	5.21	1.12	0.58

ND* not determined due to a partial overlap with another peak

### Experiments with liposomes from tissue extracts

The same experiment was performed with liposomes prepared from tissue extracts, either from the bovine liver or the bovine heart. The particle size distribution of these liposomes can be seen in the Electronic Supplementary Material Fig. S2a. The figure shows the number size distribution (based on the number of particles of the given size, i.e., each particle has the same weight regardless of its size) and the volume size distribution (based on the total volume of particles of the given size, i.e., larger particles have higher weight in the distribution). The graph shows liposome sizes ranging from 90 to 400 nm with a number mean of around 100 nm and a volume mean of around 200 nm. This shows that two populations of liposomes are present: firstly, small liposomes around 100 nm which are thermodynamically preferred size, and secondly, liposomes between 200 and 400 nm which were made during the extrusion process from bigger liposomes. Furthermore, the liposome zeta potential was measured at different pH (see Electronic Supplementary Material Fig. S2b). Liposomes from both extracts were strongly negatively charged with zeta potential values ranging from − 65 to − 33 mV, consistently obtaining slightly more negative values for the heart extract liposomes. The zeta potential of liposomes is discussed later in detail in the respective section about the effect of pH. The electropherograms obtained for ambroxol hydrochloride, maraviroc, canagliflozin, and deferasirox with the increasing amount of the tissue extract liposomes present in the BGE consisting of 10 mM sodium phosphate buffer; pH 7.10 can be seen in Electronic Supplementary Material, Fig. S3. The effective mobility and USP tailing factor values relevant to the discussion are provided in Table 2. As previously, canagliflozin (peak no. 3) migrated out of the neutral zone; however, its anodic effective mobility was in both cases roughly one order of magnitude higher than in the case of DPPC-DPPG liposomes, with a more pronounced increase in the liver-based liposomes. An interesting difference was observed also in peak symmetry. While in the DPPC-DPPG liposomes, the canagliflozin peak was relatively symmetrical (USP tailing 1.01 to 1.24), it was fronting in the BGE containing liver extract liposomes (USP tailing 0.68 to 0.91). Surprisingly, the peak was getting more symmetrical with the increasing amount of liposomes. On the other hand, in BGE with heart extract liposomes, canagliflozin peak was tailing without a significant difference in the USP tailing factor value (1.93 to 2.00). From Fig. S3, it can be seen that although the symmetry was roughly constant, the peak was broadening with the increasing amount of liposomes. Both tissue extract-based liposomes thus exhibited significantly stronger interactions with canagliflozin, which gained considerably higher anodic mobility. Differences between the lipid composition of both extracts (see Electronic Supplementary Material, Table S1) caused liver extract liposomes to influence mobility more than the heart extract liposomes. This difference was strongly manifested by the canagliflozin peak fronting in the BGE with liver extract liposomes and tailing in the BGE with heart extract liposomes. A striking difference in the interactions with liposomes from heart and liver extracts was observed for maraviroc (peak no. 2). Similarly to the DPPC-DPPG liposomes, the maraviroc peak was tailing, and the USP tailing factor was increasing with the increasing amount of liver/heart extract liposomes in BGE. Concerning the cathodic effective mobility of this API, both extract-based liposomes showed a stronger impact on the mobility than DPPC-DPPG liposomes. Going from 0 to 10% (*v*/*v*) of liposomes in BGE, the effective mobility decreased to 90% of its original value with heart extract liposomes and to roughly 20% with liver extract liposomes. Ambroxol hydrochloride (peak no. 1) lost its cathodic mobility when liver-based liposomes were added to the BGE, indicating strong interactions with these liposomes. A similar observation was made with heart-based liposomes, although a higher liposome concentration in the BGE was needed for the API to lose its mobility. In heart-based liposomes, the USP tailing factor increased 4.1 times with the addition of only 2% (*v/v*) liposomes to the BGE. For deferasirox (peak no. 4), once again, the presence of liposomes had no distinct effect on tailing factor values regardless of whether they were prepared from liver or heart extract. Anodic effective mobility of this negatively charged API slightly increased (by around 6%) in the presence of liver extract liposomes, an even smaller increase (roughly 2.5%) was observed for heart extract liposomes. As discussed above, the weak effect of liposomes on the mobility of negatively charged deferasirox can be caused either by its weak interaction with liposomes possibly hindered by their electrostatic repulsion or by the anodic effective mobility of both, API and liposomes. However, when deferasirox was measured without the presence of other APIs, its peak shape changed from fronting to tailing, suggesting that the liposomes might be saturated by other APIs in the mixture. These results suggest that the interactions between APIs and liposomes are strongly influenced by the lipid composition and charge of the liposomes. Liver-based liposomes, with their higher content of negatively charged components, exhibit stronger electrostatic interactions with certain APIs, leading to more significant changes in mobility and tailing factors. The differences observed in maraviroc behavior indicate that the specific lipid components of liver and heart extracts affect their interaction dynamics. The saturation of liposomes by other APIs in the mixture can also impact the observed peak shapes and mobilities, as seen with deferasirox. Overall, these findings highlight the importance of considering lipid composition and thus their different properties when evaluating API-liposome interactions in CE experiments.

**Table 2:** Obtained electrophoretic mobilities *μ*_eff_ and USP tailing factor in BGE containing liposomes from tissue extracts at total lipid concentration 5 mg/ml. BGE: 10 mM sodium phosphate buffer, pH 7.10 with increasing amount of liposomes; temperature: 25 °C

% lip	*μ*_eff_ ⋅ 10^8^, m^2^ V^−1^ s^−1^				USP tailing (at 5% height)			
	Ambroxol	Maraviroc	Canagliflozin	Deferasirox	Ambroxol	Maraviroc	Canagliflozin	Deferasirox
Liver extract								
0	1.56 ± 0.02	0.95 ± 0.01	-	− 1.70 ± 0.02	2.97	3.19	-	0.64
2	-	0.83 ± 0.01	− 1.78 ± 0.00	− 1.70 ± 0.01	-	3.52	ND*	0.68
4	-	0.62 ± 0.01	− 2.51 ± 0.01	− 1.73 ± 0.00	-	ND*	0.68	0.59
6	-	0.49 ± 0.01	− 2.83 ± 0.01	− 1.75 ± 0.00	-	ND*	0.76	0.58
8	-	0.31 ± 0.00	− 3.12 ± 0.01	− 1.79 ± 0.00	-	ND*	0.84	0.61
10	-	0.17 ± 0.02	− 3.30 ± 0.03	− 1.81 ± 0.01	-	ND*	0.91	0.67
Heart extract								
0	1.60 ± 0.01	1.00 ± 0.01	-	− 1.71 ± 0.01	3.10	3.63	-	0.55
2	1.53 ± 0.01	0.99 ± 0.02	− 0.75 ± 0.01	− 1.71 ± 0.00	11.6	4.79	1.98	0.55
4	1.37 ± 0.02	0.97 ± 0.01	− 1.38 ± 0.02	− 1.72 ± 0.00	12.6	4.88	1.92	0.55
6	1.14 ± 0.03	0.95 ± 0.02	− 1.88 ± 0.02	− 1.73 ± 0.00	ND*	5.45	1.87	0.56
8	-	0.95 ± 0.01	− 2.26 ± 0.03	− 1.74 ± 0.00	-	6.42	2.00	0.57
10	-	0.90 ± 0.02	− 2.58 ± 0.03	− 1.75 ± 0.00	-	8.00	1.93	0.59

ND* not determined due to a partial overlap with another peak

### Effect of temperature

**Fig. 1:**
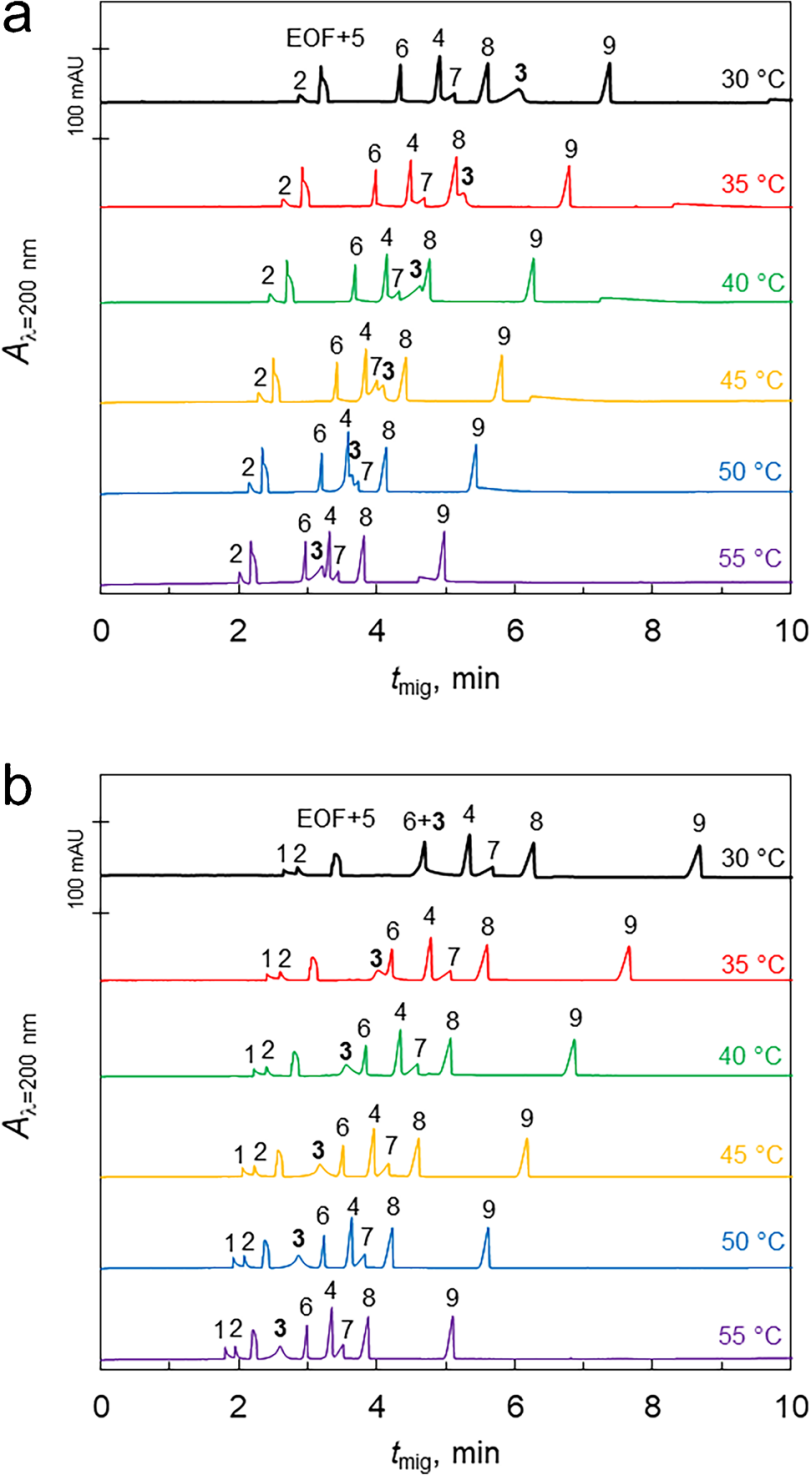
Experiment of API-liposome interactions with increasing temperature during measurements. BGE: 10 mM sodium phosphate buffer at pH 7.10 with 4% of liposomes from bovine liver (**a**) or bovine heart (**b**) extracts; API mixture contains ambroxol hydrochloride (1), maraviroc (2), canagliflozin (3), deferasirox (4), aprepitant (5), atorvastatin calcium (6), febuxostat (7), ibuprofen (8), and valsartan (9) at 0.1 mg/ml concentration

**Fig. 2:**
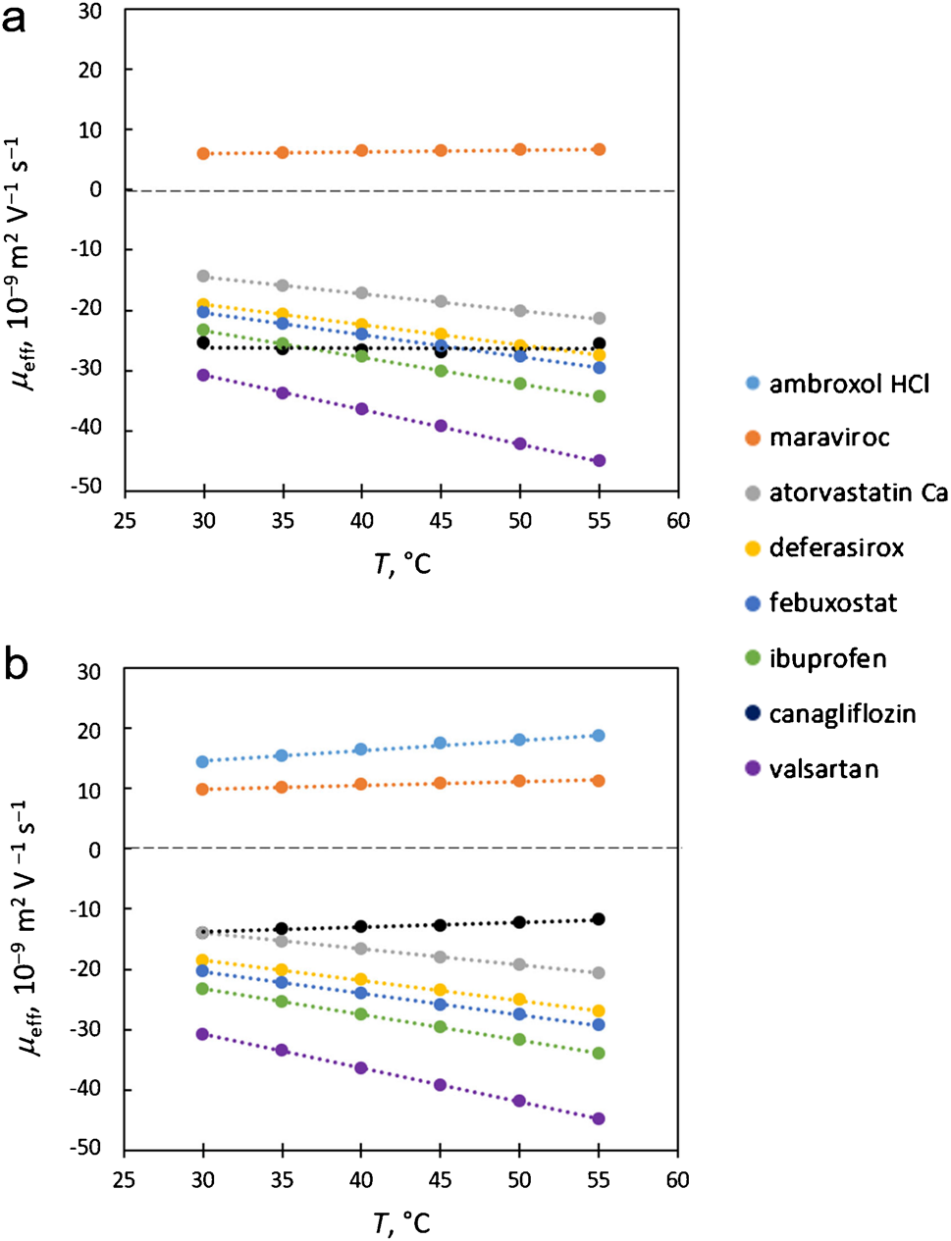
Effective mobilities of individual APIs with increasing temperature during measurements. BGE: 10 mM sodium phosphate buffer at pH 7.10 with 4% of liposomes from bovine liver (**a**) or bovine heart (**b**) extracts; API mixture contains ambroxol hydrochloride, maraviroc, canagliflozin, deferasirox, aprepitant, atorvastatin calcium, febuxostat, ibuprofen, and valsartan at 0.1 mg/ml concentration

We tested the influence of increasing temperature in a range including the physiological temperature region on API-liposome interactions for nine lipophilic APIs, namely ambroxol hydrochloride, maraviroc, canagliflozin, deferasirox, aprepitant, atorvastatin calcium, febuxostat, ibuprofen, and valsartan. As the temperature rises, the flowability of the lipid bilayer increases, which should affect these interactions. Based on previous experiments, we added 4% of either bovine liver extract or bovine heart extract liposomes to a 10 mM sodium phosphate buffer at pH 7.10 and used these mixtures as BGE for the separation of all nine APIs. Electropherograms obtained with capillary cassette temperature ranging from 30 to 55 °C for bovine liver and bovine heart extract liposomes are shown in Fig. 1a and b, respectively. Migration times of all observed peaks are significantly decreasing with increasing temperature, which can be attributed to the decreasing viscosity of the BGE which is strongly influenced by the temperature. There are no significant changes in the peak shapes observed as a result of the changing temperature. For further discussion, it is important to consider the changes in the effective mobility of the APIs rather than absolute migration times. We thus calculated the effective mobilities of the APIs and plotted them against the temperature (Fig. 2). We observed that the effective mobility increased linearly with temperature for most APIs, which can be attributed not only to the lower viscosity of the BGE but also to the enhanced fluidity of the liposomal membrane. This makes it easier for the APIs to migrate through the capillary. However, canagliflozin (peak no. 3) exhibited different behavior. In liver-based liposomes, its anodic effective mobility remained almost unchanged, while in heart-based liposomes, its effective mobility decreased, in contrast to all other tested APIs. This suggests that canagliflozin has a lower affinity for liposomes as the lipid bilayer becomes less rigid with increasing temperature. The unique behavior of canagliflozin indicates that its interaction with liposomes is particularly sensitive to changes in the lipid bilayer rigidity as a significant reduction in interaction strength can be observed at higher temperatures. To compare the behavior of canagliflozin with another uncharged API, we added aprepitant (API no. 5) into the mixture. However, the aprepitant exhibited zero electrophoretic mobility throughout all our experiments, suggesting no ongoing interactions with our liposomes. This observation further points out the complexity of the API-liposome interactions. Aprepitant exhibits the third highest log P value of the test set of API, notably significantly higher than strongly interacting canagliflozin (see Table S2), and yet, no interaction with liposomes was observed. Overall, this experiment highlights the importance of considering temperature effects when evaluating API-liposome interactions as these effects can significantly differ for individual APIs. The differences in behavior between liver-based and heart-based liposomes also underscore the role of lipid composition in these interactions. Understanding these variables can help optimize liposome-based drug delivery systems, ensuring effective drug encapsulation and release under physiological conditions.

### Effect of pH

**Fig. 3:**
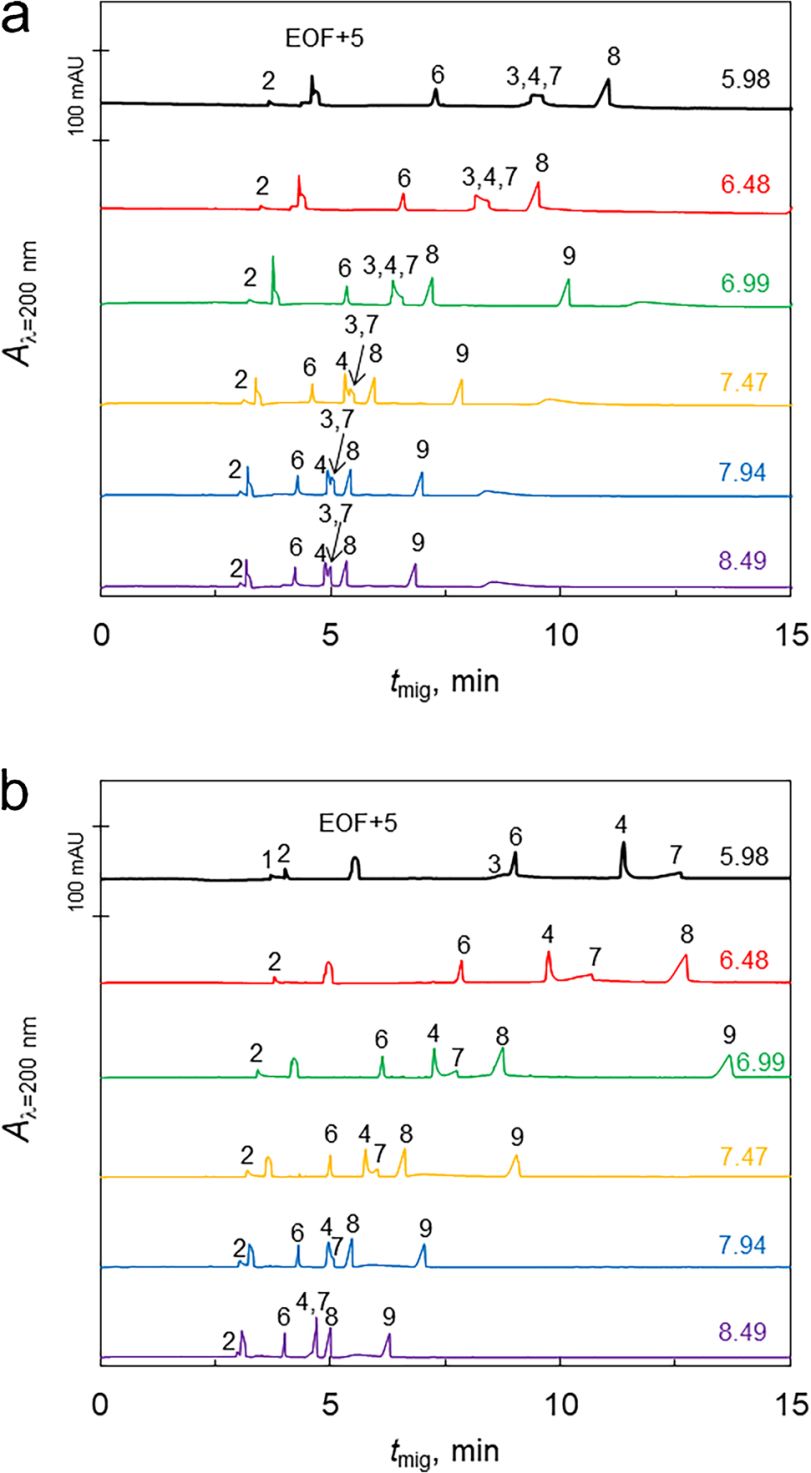
Experiment of API-liposome interactions with increasing pH of the BGE. BGE: sodium phosphate buffer at constant ionic strength (I = 20 mM) with 4% of liposomes from bovine liver (**a**) or bovine heart (**b**) extracts. API mixture contains ambroxol hydrochloride (1), maraviroc (2), canagliflozin (3), deferasirox (4), aprepitant (5), atorvastatin calcium (6), febuxostat (7), ibuprofen (8), and valsartan (9) at 0.1 mg/ml concentration

**Fig. 4:**
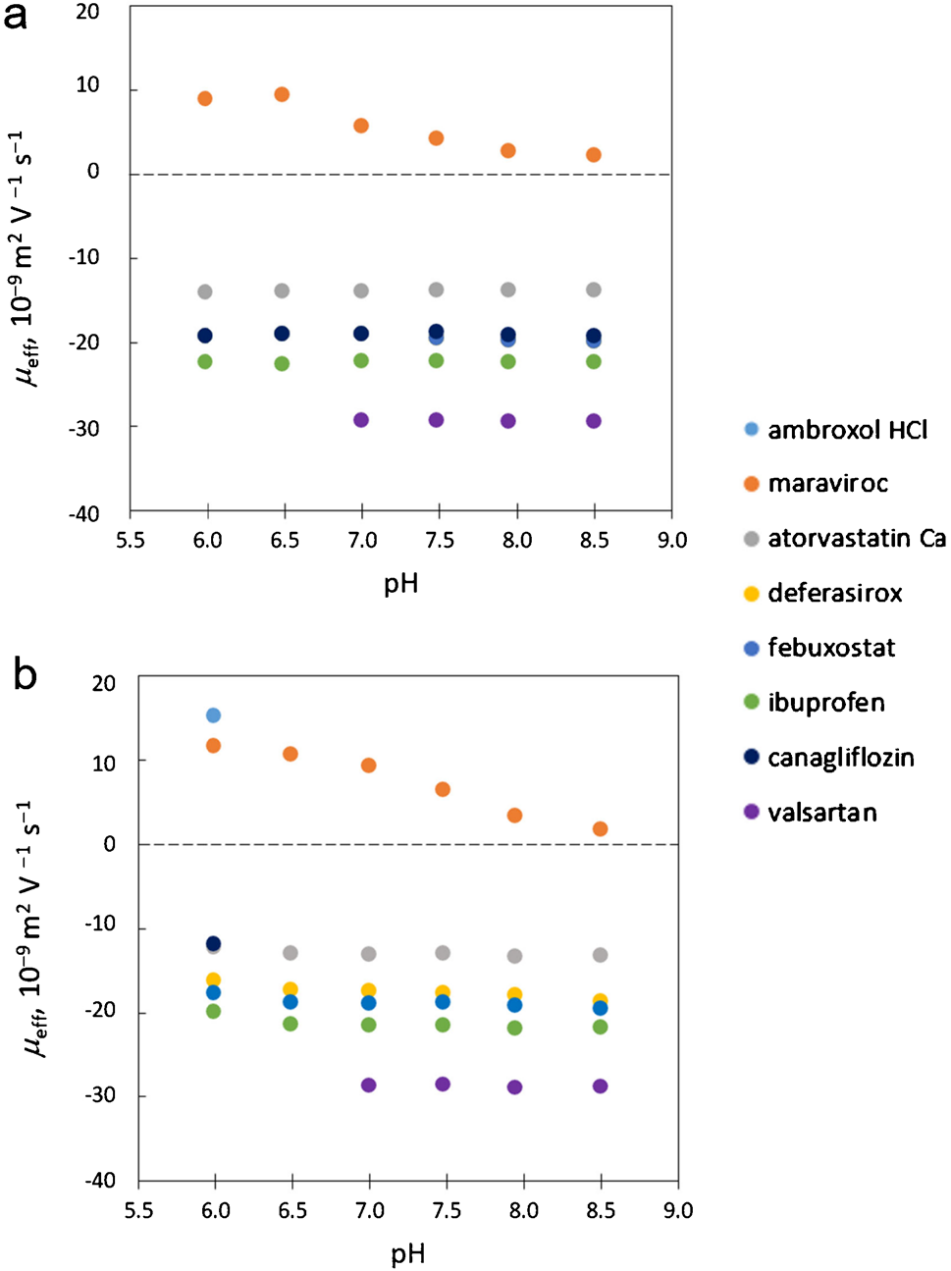
Effective mobilities of individual APIs with increasing pH during measurements. BGE: 10 mM sodium phosphate buffer with 4% of liposomes from bovine liver (**a**) or bovine heart (**b**) extracts; temperature: 25 °C. API mixture contains ambroxol hydrochloride, maraviroc, canagliflozin, deferasirox, aprepitant, atorvastatin calcium, febuxostat, ibuprofen, and valsartan at 0.1 mg/ml concentration

Lastly, we tested how the different pH levels might influence API-liposome interactions, considering that some APIs might exhibit pH sensitivity. To mimic the physiological conditions, we selected a pH range representing the variability observed across different tissues. We prepared six sodium phosphate buffers with an identical ionic strength of 20 mmol/l, with pH values ranging from 6.0 to 8.0 in 0.5 increments. We added 4% of either bovine liver or bovine heart extract liposomes to these buffers and used them as BGEs for the separation of the nine test APIs. Electropherograms of these separations under different pH for bovine liver and bovine heart extract liposomes are shown in Fig. 3a and b, respectively. Increasing pH of the BGE resulted in decreasing migration times of the APIs which can be explained by the increasing mobility of EOF which is strongly affected by the pH of BGE. To discuss other effects of the pH, it is necessary to consider effective mobilities of APIs. The effective mobility values plotted as a function of pH are shown in Fig. 4. Zeta potential of the liposomes was measured at these pH (see Electronic Supplementary Material Fig. S2b), and it was shown that both liver and heart extract liposomes are negatively charged at all pH, going slightly more negative with higher pH and that the heart extract liposomes are always more negative than the ones from liver extract. When the results are shown as pH-dependent mobilities (Fig. 4) for molecules with negative mobility, the pH dependency is weak or non-existent. This indicates that most of the effect on migration times seen in Fig. 3 is caused by the influence of pH on the EOF. The only strong dependency of electrophoretic mobility on pH showed maraviroc for both liver and heart extract. The effective mobility of this API declines almost to zero between pH 6.0 and 8.0. Such behavior can, in principle, be caused by deprotonation of the positively charged API. Nevertheless, this is not the case as the pKa of maraviroc is well above the tested pH range (Table S2). This effect thus can be attributed to a significant change in the maraviroc-liposome interactions taking place within this pH range. It turned out to be challenging to distinguish whether the observed changes in API behavior were due to the variations in API charge at different pH levels or if the presence of liposomes also contributed to these changes. The influence of pH on API-liposome interactions is a complex phenomenon, as both the charge of the APIs and the liposomal membrane can be affected by pH changes. APIs can ionize differently at various pH levels, altering their charge and subsequently their interaction with the liposomes. Similarly, the surface charge of the liposomes can change with pH, influencing the electrostatic interactions between the APIs and the liposomal membrane. In our experiments, the difficulty in distinguishing the effects of pH changes from the effects of the presence of liposomes suggests that both factors likely play significant roles in modifying API behavior. The only molecule for which a strong influence on pH was shown was maraviroc. Here, the molecular properties changing with pH play a strong role because the change of electrophoretic mobility is much stronger than the change of liposome charge through the investigated pH range.

## Conclusion

We explored the interactions between various APIs and liposomes used as a pseudostationary phase in liposomal electrokinetic chromatography. Our preliminary experiments demonstrated that liposomes prepared from DPPC and DPPG lipids can influence the separation behavior of APIs. For subsequent experiments, unlike most studies to date, we used liposomes prepared from complex tissue extracts rather than simple lipid mixtures. Experiments with liposomes derived from bovine liver and heart tissue extracts revealed distinct interaction patterns. Effective mobilities of neutral canagliflozin, positive maraviroc, and ambroxol as well as negative deferasirox showed stronger interactions with liver-based liposomes, attributed to their higher negative charge and different lipid composition. Changes in peak shapes displayed varied interaction behaviors between liver-based and heart-based liposomes, highlighting the role of lipid composition in API-liposome interactions. Notably, deferasirox peak shape changed when measured alone, suggesting potential saturation effects in the presence of other APIs. Temperature studies highlighted that increasing the temperature generally enhanced the effective mobility of most APIs, due to lower BGE viscosity and increased liposomal membrane fluidity. However, canagliflozin mobility decreased at higher temperatures, particularly in heart-based liposomes, indicating a reduced affinity as the lipid bilayer became less rigid. This suggests that canagliflozin interaction with liposomes is sensitive to changes in the bilayer rigidity. Lastly, pH studies revealed the complexity of the effects of pH and liposome presence on API behavior. Changes in API charge at different pH levels and the corresponding alterations in liposomal surface charge both play significant roles in API-liposome interactions. We did not observe any straightforward effect of hydrophobicity or charge of the API on its interactions with liposomes, which further underlines the complexity of the interactions that must be taken into consideration. The only exception was the generally observed weaker effect on mobility of negatively charged APIs. Overall, our findings underscore the importance of considering lipid composition, temperature, and pH when evaluating API-liposome interactions in CE experiments. These factors significantly impact the behavior of APIs and can influence the optimization of liposome-based drug delivery systems for enhanced drug encapsulation, release, and targeting under physiological conditions.

## Supplementary Information

Below is the link to the electronic supplementary material.

Supplementary file1 (DOCX 262 KB)

## Abbreviations

API: Active pharmaceutical ingredient
BGE: Background electrolyte
CE: Capillary electrophoresis
CEC: Capillary electrochromatography
DPPC: 1,2-Dipalmitoyl-*sn*-glycero-3-phosphocholine
DPPG: 1,2-Dipalmitoyl-*sn*-glycero-3-phospho-(1′-*rac*-glycerol) (sodium salt)
EOF: Electroosmotic flow
LEKC: Liposomal electrokinetic chromatography
