# Supplementary material for: Investigating drug-liposome interactions using liposomal electrokinetic chromatography
Source: Anal Bioanal Chem. 2025 Feb 13;417(10):2029–38. doi: 10.1007/s00216-025-05783-6 (PMC11961511; doi:10.1007/s00216-025-05783-6)
Supplement: Supplementary file 1 — Supplementary file1 (DOCX 262 KB) [file 216_2025_5783_MOESM1_ESM.docx]

**Electronic Supplementary Material**

**Investigating Drug-Liposome Interactions Using Liposomal Electrokinetic Chromatography**

Alice Šimonová^1,2^, Martin Balouch^2,3^, František Štěpánek^3^, Tomáš Křížek^1^

____

tomas.krizek@natur.cuni.cz

^1^ Faculty of Science, Department of Analytical Chemistry, Charles University, Hlavova 8, Prague 2, 128 00, Czech Republic

^2^ Zentiva, k.s., U Kabelovny 130, Prague 10, 102 37, Czech Republic

^3^ University of Chemistry and Technology, Department of Chemical Engineering, Technická 5, Prague 6, 166 28 Czech Republic

**Table S1** – Lipid composition of individual extracts*, PC – phosphatidylcholine,
PE – phosphatidylethanolamine, PI – phosphatidylinositol, PA – phosphatidic acid, CA – cardiolipin

| Heart extract (bovine) | | Liver extract (bovine) | |
| --- | --- | --- | --- |
| Component | wt/wt% | Component | wt/wt% |
| PC | 8.6 | PC | 42 |
| PE | 13.6 | PE | 26 |
| PI | 1.0 | PI | 9 |
| PA | 0.6 | Lyso PI | 1 |
| CA | 1.7 | Cholesterol | 5 |
| Neutral lipid | 57.7 | Others (neutral lipids) | 17 |
| Unknown | 16.8 |  |  |

* Lipid profile of Liver Extract Polar (Bovine) and Heart Extract Polar (Bovine). Avanti Polar Lipids, Inc. Retrieved November 10, 2023, from <https://avantilipids.com/>

**Table S2** – Used APIs and their characteristics

| No. | API | *pKa** | log *P** | charge at pH 7.10 |
| --- | --- | --- | --- | --- |
| 1 | Ambroxol HCl | 15.26 | 2.65 | + |
| 2 | Maraviroc | 13.98 | 3.63 | + |
| 3 | Canagliflozin | 12.57 | 3.52 | n |
| 4 | Deferasirox | 4.51 | 4.74 | - |
| 5 | Aprepitant | 6.59 | 5.22 | n |
| 6 | Atorvastatin Ca | 4.31 | 5.39 | - |
| 7 | Febuxostat | 3.08 | 3.52 | - |
| 8 | Ibuprofen | 4.85 | 3.84 | - |
| 9 | Valsartan | 4.35 | 5.27 | - |

*Data for individual APIs obtained from DrugBank. Accessed June 13, 2024, from <https://www.drugbank.com/>


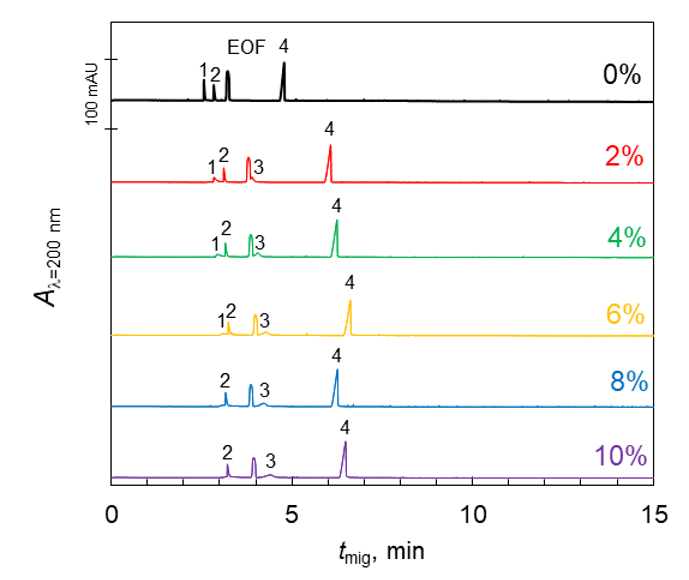


**Fig. S1** – Preliminary experiment of API-liposome interactions;
BGE: 10 mM sodium phosphate buffer at pH 7.10 with increasing amount of DPPC:DPPG (3:1) liposomes; API mixture contains ambroxol hydrochloride (1), maraviroc (2), canagliflozin (3) and deferasirox (4) at 0.1 mg/ml concentration


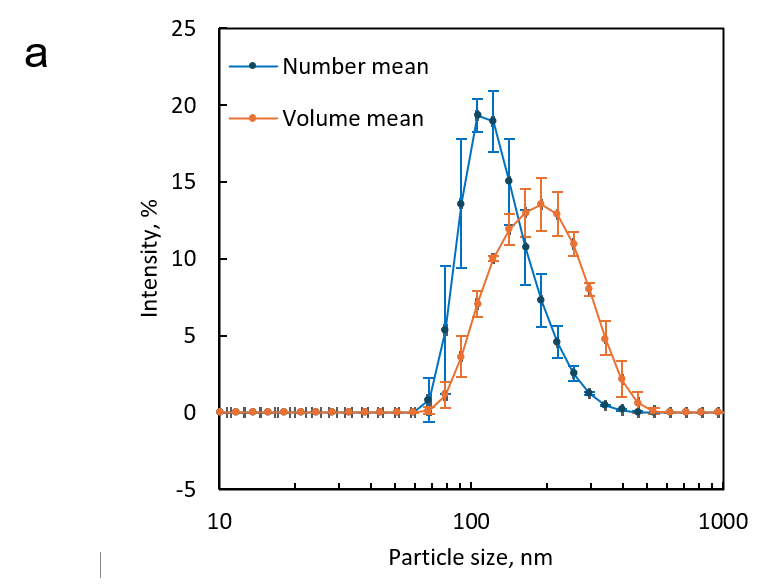


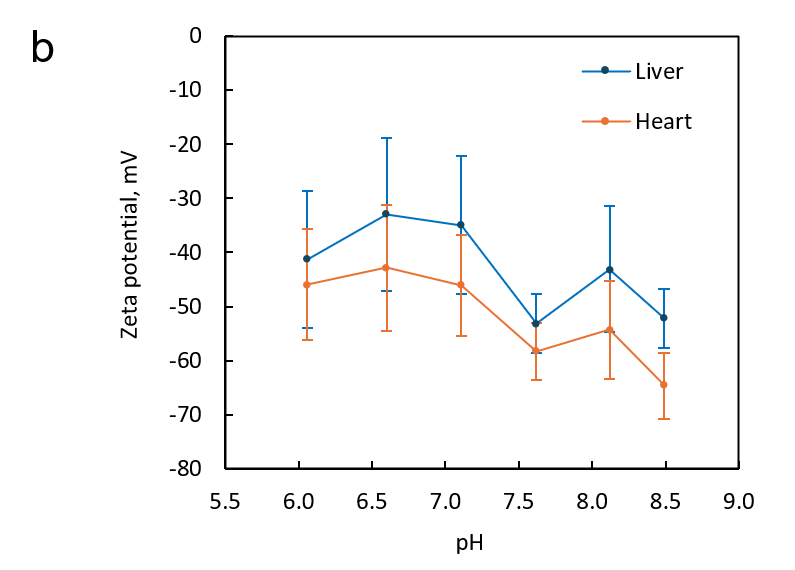


**Fig. S2** – Characterisation of liposomes prepared from liver and heart extracts. Particle size distribution of produced liposomes, data from Liver extract at 7.1 pH **(a)**. Zeta potential dependence on pH for liver and heart extract **(b).**


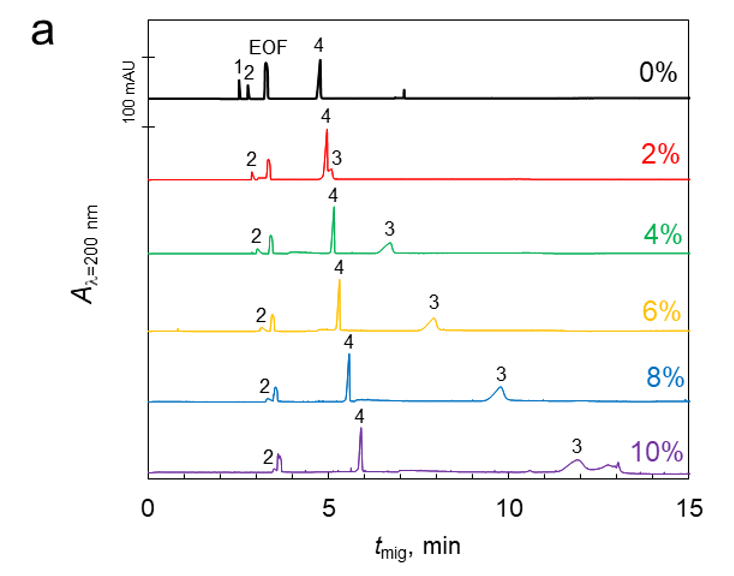

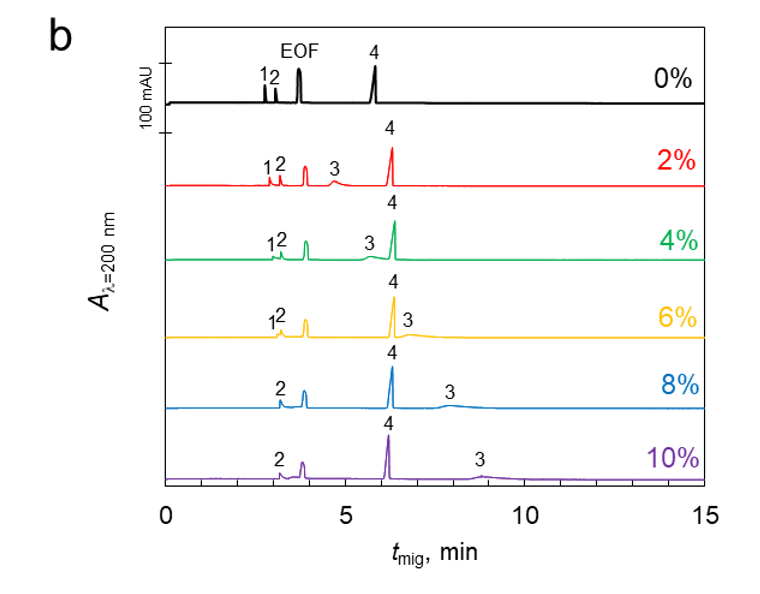


**Fig. S3** – Experiment of API-liposome interactions;
BGE: 10 mM sodium phosphate buffer at pH 7.10 with increasing amount of liposomes from bovine liver **(a)** or bovine heart **(b)** extracts; API mixture contains ambroxol hydrochloride (1), maraviroc (2), canagliflozin (3) and deferasirox (4) at 0.1 mg/ml concentration
